# Effective Small Interfering RNA Therapy to Treat *CLCN7*-dependent Autosomal Dominant Osteopetrosis Type 2

**DOI:** 10.1038/mtna.2015.21

**Published:** 2015-09-01

**Authors:** Mattia Capulli, Antonio Maurizi, Luca Ventura, Nadia Rucci, Anna Teti

**Affiliations:** 1 Department of Biotechnological and Applied Clinical Sciences, University of L'Aquila, L'Aquila, Italy; 2 Department of Pathology, San Salvatore Hospital, L'Aquila, Italy

**Keywords:** bone disease, bone resorption, gene silencing, osteoclast, osteopetrosis, siRNA therapy

## Abstract

In about 70% of patients affected by autosomal dominant osteopetrosis type 2 (ADO2), osteoclast activity is reduced by heterozygous mutations of the *CLCN7* gene, encoding the ClC-7 chloride/hydrogen antiporter. *CLCN7*^*G215R*^-, *CLCN7*^*R767W*^-, and *CLCN7*^*R286W*^-specific siRNAs silenced transfected mutant mRNA/*EGFP* in HEK293 cells, in RAW264.7 cells and in human osteoclasts, with no change of *CLCN7*^*WT*^ mRNA and no effect of scrambled siRNA on the mutant transcripts. Osteoclasts from *Clcn7*^*G213R*^ ADO2 mice showed reduced bone resorption, a condition rescued by *Clcn7*^G213R^-specific siRNA. Treatment of ADO2 mice with *Clcn7*^*G213R*^-specific siRNA induced increase of bone resorption variables and decrease of trabecular bone mass, leading to an overall improvement of the osteopetrotic bone phenotype. Treatment did not induce overt adverse effects and was effective also with siRNAs specific for other mutants. These results demonstrate that a siRNA-based experimental treatment of ADO2 is feasible, and underscore a translational impact for future strategy to cure this therapeutically neglected form of osteopetrosis.

## Introduction

Autosomal dominant diseases are often characterized by severe morbidity and lack of effective treatments.^1,2,3^ Consistent with the dominant negative nature of these genetic conditions, they are fairly good candidates for mutant gene silencing by RNA interference.^4,5^

RNA interference is a mechanism by which gene expression is regulated at the post-transcriptional level.^6^ It relies on the ability of cells to transcribe and process small RNAs that complement specific mRNA sequences, inducing their degradation.^6,7^

Experimental RNA interfering occurs by two principal molecules, the small hairpin (sh)RNAs and the small interfering (si)RNAs.^8^ To silence an mRNA by shRNA, transfection vectors must be used to transduce cells, which will then express and process the shRNA to make it active in gene silencing.^9^ From a therapeutic point of view, shRNA delivery requires approaches similar to gene therapy, which would limit its use especially to local diseases.^9^ siRNA are instead double-strand 15 to 25 nucleotide sequences that freely influx the cells.^10^ Transfection agents may improve the efficiency of siRNA delivery, but especially for primary cells, they are not always strictly necessary.^11^ siRNAs are generally highly specific and efficient in silencing gene expression^12^ and, therapeutically, they are gaining great interest for the theoretical possibility to be used in clinic to treat systemic conditions, such as those caused by neoplastic somatic mutations^13^ or by germline dominant negative genetic alterations.^14^ siRNA sequences can be designed to complement a given mRNA in a highly specific and efficient manner,^15^ with a certain degree of serendipity still necessary to obtain effective gene silencing.^12,15^

Especially in cancer, siRNAs have been found to be suitable for targeted therapies in animal models, and clinical trials are currently being developed to eliminate deregulated pathways that transform cells and make them aggressive.^16,17,18^ Recent work pointed to the use of siRNAs also in osteoporosis, especially targeting pathways implicated in osteoclastogenesis and bone resorption.^19^ Various modifications are being tested to improve stability, pharmacokinetics, delivery reproducibility, and tissue distribution.^16,19^ In addition, diverse vehicles have been found to improve stability and availability, concurring to optimize the use *in vivo* and minimize siRNA immune and inflammatory responses.^13,20,21^ The number of diseases hypothesized to be cured by siRNA therapy, including autosomal dominant genetic disorders, is steadily increasing. It has been observed that siRNAs can distinguish mRNA species also by a single divergent nucleotide,^22^ and there are means to increase specificity,^23^ with the result that only the mutant mRNA undergoes degradation, with no effect on the normal mRNA.^24^

Autosomal dominant osteopetrosis type 2 (ADO2), or Albers-Schönberg disease,^25^ is a genetic bone condition due, in roughly 70% of patients, to heterozygous missense mutations of the *CLCN7* gene. This gene encodes the ClC-7 protein, formerly considered a chloride channel (type 7),^26^ but now reclassified as a chloride/hydrogen antiporter.^27^ ClC-7 is expressed in various organs, including bone, liver, kidney, heart, spleen, and brain,^26^ but its mutations greatly affect especially bone and brain. *CLCN7* loss-of-function mutations lead to autosomal recessive osteopetrosis, characterized by severe skeletal and, often, cognitive phenotype.^28^ In bone, stunted growth, increased bone mass, and constrain of the bone marrow cavities are observed along with extreme bone fragility, hematological failure, recurrent infections, and osteomyelitis.

In the bone tissue, the mutation affects the osteoclasts, multinucleated cells that disrupt the bone matrix through the acidification of the extracellular space confined between the osteoclast plasma membrane and the bone surface, called resorption lacuna.^29^ Here protons are released through a vacuolar H^+^-ATPase and this release is charge balanced by the ClC-7 protein that discharges chloride ions into the resorption lacuna. Neural impairments, typically due in autosomal recessive osteopetrosis to nerve compression syndromes, in this form can be aggravated by primary hippocampal, cortical and retinal degeneration caused by lysosomal storage disease.^30^ The *CLCN7* gene is aplosufficient, and single allele loss of function mutant carriers display no symptoms whatsoever.^31,32^

At variance with autosomal recessive osteopetrosis, *CLCN7*-dependent ADO2 is not life threatening but can be seriously debilitating. It presents with dense bones, especially at skull base, pelvis, and vertebras. 66% of the patients show disease symptoms, which range from mild to severe, very rarely lethal,^3,31^ with the remaining patients representing unaffected carriers.^31^ Along with high bone mass, patients display bone fragility that, in the most severe cases, can cause tens of nontraumatic fractures, very difficult to be treated surgically. Osteomyelitis, reduced vision and hearing due to nerve compression, infections and some hematological failures are also observed in severe forms, while cognitive functions are generally preserved.^32,33^

*CLCN7*-dependent ADO2 has no cure. Palliative interventions to control pain, reduce fractures, decompress nerves, and improve hematological symptoms are used, with albeit poor effectiveness.^31^ Therefore, there is an urgent need to identify efficient methods to treat this therapeutically neglected disease. In this work, we hypothesized that *CLCN7*^*mutant*^-specific siRNAs could be effective in silencing dominant negative *CLCN7* transcripts *in vitro* and *in vivo*, representing an efficacious means to ameliorate the bone phenotype in ADO2. We tested our siRNA in an animal model of ADO2 (ref. 34) and found a treatment regimen that increased bone resorption and reduced bone mass, rescuing the bone phenotype to normal levels.

## Results

### Set-up of HEK293 cell models

**Figure 1:**
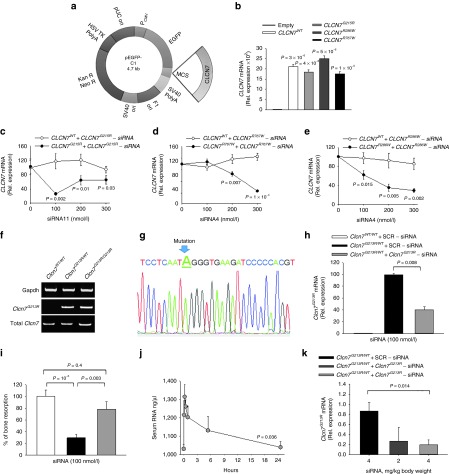
***In vitro* and *in vivo* tests of *CLCN7*^*mutant*^-specific siRNAs**. (**a**) Cartoon depicting the pEGFP-C1 vector used in the study. (**b**) HEK293 cells stably transfected with the pEGFP-C1 vector carrying the indicated mutations. Expression of the *CLCN7* gene was quantified by real-time RT-PCR on RNA extracted from mutant transfectants, against cells transfected with the empty vector, which did not express *CLCN7* mRNA (first bar from left). (**c–e**) HEK293 cells transfected with the indicated vectors, were treated with the *CLCN7*^*mutant*^-specific siRNA listed in Table 2 as the most effective per each mutation. Concentration-dependent regulation of *CLCN7* assessed by real-time RT-PCR, normalized with *GAPDH*. (**f**) RT-PCR using primer pairs specific for the *Clcn7*^*G213R*^ mRNA showing transcript amplification only in heterozygous (*Clcn7*^*G213R/WT*^) and homozygous (*Clcn7*^*G213R/G213R*^) osteoclasts, while in wild-type osteoclasts (*Clcn7*^*WT/WT*^) no transcript was amplified. (**g**) Direct DNA sequencing of the amplified transcript shown in **f** for the *Clcn7*^*G213R/WT*^ osteoclasts, demonstrating only the mutant sequence. (**h**) Osteoclasts generated from the bone marrow mononuclear cells of *Clcn7*^*WT/WT*^ and *Clcn7*^*G213R/WT*^ mice were treated with the indicated concentration of scrambled (SCR) or *Clcn7*^*G213R*^-specific siRNA. Real-time RT-PCR was performed using the primer pairs specific for the mutant transcript validated in (**f**) and (**g**). (**i**) Osteoclasts were generated from the bone marrow mononuclear cells of *Clcn7*^*WT/WT*^ and *Clcn7*^*G213R/WT*^ mice onto bone slices and treated with the indicated concentration of SCR and *Clcn7*^*G213R*^-specific siRNA. At the end of experiment, cells were removed by sonication and bone resorption evaluated by the pit assay. (**j**) Three-month-old *Clcn7*^*WT/WT*^ mice were injected once i.p. with 4 mg/kg of *Clcn7*^*G213R*^-sticky siRNA jetPEI conjugate and sacrificed at the indicated time point. Sera were collected and evaluated for total RNA concentration by Nanodrop. (**k**) Ten-day-old *Clcn7*^*G21R/WT*^ mice were injected once i.p. with the indicated doses of SCR- or of *Clcn7*^*G213R*^-sticky siRNA jetPEI conjugate. After 48 hours, mice were sacrificed, RNA was extracted from tibias, and evaluated by real-time RT-PCR using the primer pairs specific for the *Clcn7*^*G213R*^ mRNA validated in (**f**) and (**g**). In **b–e, h–k** data are the mean ± SD of three independent experiments or three animals/group. **b–e,h,I,k**: Student's *t*-test. **j**: one-way analysis of variance (ANOVA). For **c–e**, statistics was also performed by one way ANOVA (shown in **Supplementary Table S3**).

In this study, we investigated three *Clcn7* mutants, *Clcn7*^*R767W*^, *Clcn7*^*R286W*^, and *Clcn7*^*G215R*^. By site-directed mutagenesis, we obtained pEGFP-C1 vectors carrying the constructs with the desired mutation tagged with the *EGFP* (**Table 1** and **Figure 1a**), that were used to transfect human HEK293 cells (**Figure 1b**–**e**), human peripheral blood mononuclear cells (see **Figure 4a**–**c**), human MDA-MB-231 cells (see **Figure 4e**), and mouse RAW264.7 cells (see **Supplementary Figure S1**).

### siRNA design and *in vitro* tests

ADO2 CLCN7 mutations are shown in **Supplementary Table S1**. A series of *CLCN7*^*mutant*^-specific siRNAs were designed for the three mutations (**Table 2** and **Supplementary Table S1**) and tested in transfected HEK293 cells for their ability to reduce *CLCN7*^*mutant*^ expression in an efficient and highly specific manner. Scrambled siRNAs were used as negative controls. We observed that most siRNAs diverging from *CLCN7*^*WT-EGFP*^ only for the mutant nucleotide, even if efficient, were not enough specific as they silenced to a similar extent also the *CLCN7*^*WT-EGFP*^ mRNA (**Table 2**). According to Onishi *et al*.,^24^ we improved the specificity between *CLCN7*^*WT-EGFP*^ and *CLCN7*^*mutant-EGFP*^ mRNA silencing by introducing additional mismatch nucleotides in various positions of the siRNAs (**Table 2**). Using our screening strategy, we selected one siRNA per each *CLCN7* mutation that was effective in silencing the *CLCN7*^*mutant-EGFP*^ in HEK293 cells without affecting the *CLCN7*^*WT-EGFP*^ (**Table 2**; **Figure 1c**–**e**). These siRNAs knocked down the *CLCN7*^*mutant-EGFP*^ transcript also in transfected RAW264.1 cells, which represent a murine model of osteoclast precursors (**Supplementary Figure S1**).

### Clcn7^G213R^ gene silencing and bone resorption tests in mouse Clcn7^G213R/WT^ ADO2 osteoclasts

To perform the *in vivo* tests, we used the *Clcn7*^*G213R/WT*^ knock-in mice recently generated in our laboratory.^34^ These mice harbor the murine homolog (G213R) of the human ClC-7 G215R amino acid substitution and show a bone phenotype similar to the human disease, thus representing a genuine model of human ADO2. To induce *Clcn7*^*G213R*^ gene silencing, we used the specific *CLCN7*^*G215R*^ siRNA effective on the human mutant gene, which displayed the mutant nucleotide and one additional mismatch nucleotide compared to the murine wild-type sequence (**Table 3**). This siRNA was first tested *ex vivo* for its efficiency in decreasing osteoclast *Clcn7*^*G213R*^ mRNA and rescuing bone resorption. To this end, bone marrow mononuclear cells were isolated from *Clcn7*^*WT/WT*^ and *Clcn7*^*G213R/WT*^ ADO2 mice and differentiated into mature osteoclasts on plastic or bone slices. In this experimental condition, using primer pairs that discriminated the mutant from the normal mRNA form (**Supplementary Table S2**, **Figure 1f**,**g**), we observed that treatment of ADO2 osteoclasts with *Clcn7*^*G213R*^-siRNA reduced the transcriptional expression of *Clcn7*^*G213R*^, without affecting the *Clcn7*^*WT*^ transcript (**Figure 1h**) nor the expression of other genes of the *Clcn* family, such as *Clcn5* and *Clcn5* (**Supplementary Figure S2**). *Clcn7*^*G213R/WT*^ ADO2 osteoclasts displayed a resorption pit formation ability of 30% of *Clcn7*^*WT/WT*^ osteoclasts (**Figure 1i**), mimicking the rate of reduced bone resorption observed in *bona fide* human ADO2 osteoclasts.^33,35^ Treatment with *Clcn7*^*G213R*^-siRNA improved bone resorption (**Figure 1i**), thus indicating an efficient rescue of activity of the *Clcn7*^*G213R/WT*^ ADO2 osteoclasts. Furthermore, although *Clcn7*^*G213R/WT*^ ADO2 cells formed more osteoclasts than *Clcn7*^*WT/WT*^ cells,^34^ equal numbers of osteoclasts were observed in *Clcn7*^*G213R/WT*^ ADO2 cultures treated with *Clcn7*^*G213R*^-specific siRNA versus scrambled siRNA-treated cells (**Supplementary Figure S3**), suggesting no effect of our treatment on osteoclast formation.

### Optimization of Clcn7^G213R^-siRNA *in vivo* delivery

For *in vivo* experiments, our *Clcn7*^*G213R*^-siRNA was conjugated with the PolyPlus Transfection jetPEI reagent, a linear polyethylenimine derivative providing effective and reproducible *in vivo* siRNA delivery, preventing inflammatory responses.^36^ To allow our *Clcn7*^*G213R*^-siRNA to efficiently conjugate with the jetPEI reagent, as suggested by the jetPEI reagent manufacturer, its sequence was modified by 3'dAdT overhangs that favor siRNA oligomerization and binding to jetPEI through these sticky ends.^37^

*Clcn7*^*G213R*^-sticky siRNA/jetPEI conjugate was then tested preliminarily in *Clcn7*^*G213R/WT*^ mice to identify optimal route of administration, dose and time of treatment. We observed that i.p. injection was to be preferred as it allowed to start the treatments already at day P10, while tail vein injection could not be performed before 1 month of age, nor did it allow frequent reiterations due to vein vessel damage.

I.p. administration of *Clcn7*^*G213R*^-sticky siRNA/jetPEI caused an increase of serum total RNA level peaking after 15 minutes, followed by a slower decline, with a return to near basal level within 24 hours (**Figure 1j**). This type of administration induced a time- and dose-dependent *Clcn7*^*G213R*^ silencing, with maximal effect by 4 mg/kg body weight observed at 48 hours (**Figure 1k**). Based on these observations, we decided to treat *Clcn7*^*G213R/WT*^ mice with 4 mg/kg body weight *Clcn7*^*G213R*^-sticky siRNA/jetPEI with a frequency of three times a week.

### *In vivo* tests

**Figure 2:**
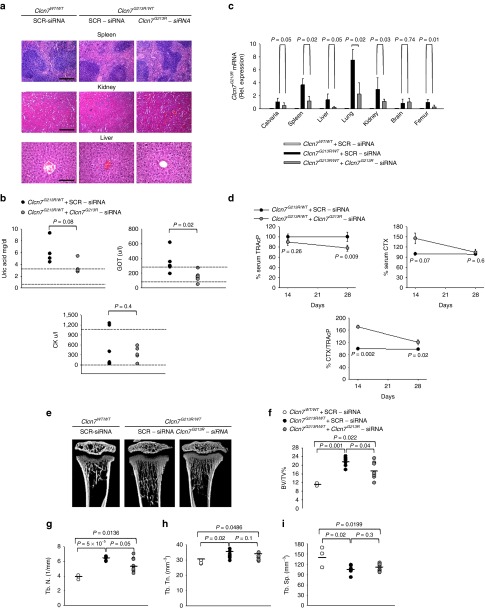
***In vivo* treatment and safety study**. Ten-day-old *Clcn7*^*G213R/WT*^ mice were injected i.p. with 4 mg/kg of *Clcn7*^*G213R*^-sticky siRNA jetPEI conjugate, three times a week for 4 weeks. At the end of the experiments, mice were sacrificed and (**a**) the indicated organs were subjected to histopathological evaluation by hematoxylin/eosin staining (Bar = 100 µm for spleen and kidney, 20 µm for liver). (**b**) Sera were collected and analyzed by the Reflotron method for the indicated biomarkers of kidney and liver disease, and for the ADO2 biomarker CK. Normal values are between the two dotted lines. (**c**) RNA was extracted from the indicated organs and subjected to real time RT-PCR using primer pairs specific for the *Clcn7*^*G213R*^ mRNA, normalized for *gapdh*. (**d**) Ten day-old *Clcn7*^*WT/WT*^ and *Clcn7*^*G213R/WT*^ were treated with 4 mg/kg of scrambled- (SRC) or *Clcn7*^*G213R*^-sticky siRNA jetPEI conjugate, three times a week for 2 and 4 weeks. At the end of the experiments, mice were sacrificed, then the serum biomarker of bone resorption, CTX, the serum osteoclast biomarker, TRAcP (5b isoform), and the CTX/TRAcP ratio were evaluated after 2 and 4 weeks of treatment. (**e**) µCT analysis of proximal tibias of mice treated with 4 mg/kg of *Clcn7*^*G213R*^-sticky siRNA jetPEI conjugate, three times a week for 2 weeks, followed by measurements of trabecular (**f**) bone volume over total tissue volume (BV/TV), (**g**) trabecular number (Th.N), (**h**) thickness (Tb.Th), and (**i**) separation (Tb.Sp). Data are (**a,e**) representative or (**b–d, f–i**) the mean ± SD of four to seven mice per group (Student's *t*-test). For **f–i** statistics was also performed by one-way analysis of variance (shown in **Supplementary Table S3**).

To assess whether *Clcn7*^*G213R*^-sticky siRNA/jetPEI could affect the ADO2 bone phenotype, we treated *Clcn7*^*G213R/WT*^ mice with our conjugate, three times a week for 2 and 4 weeks. During the treatment, mice appeared healthy and active, with no signs of distress. At autopsy, we observed no gross alteration of visceral organs, confirmed also histopathologically (**Figure 2a**), and an improvement of serum biomarkers of kidney and liver diseases, along with a reduction of serum creatine kinase (**Figure 2b**). The *CLCN7* transcript has a wide cellular distribution in human and mouse (**Supplementary Figure S4**), and in bone it is highly expressed especially in osteoclasts (**Supplementary Figures S4b,c and S5**). Real-time reverse transcriptase-polymerase chain reaction (RT-PCR) confirmed that *Clcn7*^*G213R*^ mRNA expression was reduced in all organs tested but the brain (**Figure 2c**), suggesting no crossing of the blood–brain barrier. Since our mice did not show obvious central nervous system alterations,^34^ and patients are generally reported not to suffer from cognitive failures,^32,33^ this circumstance should have no important consequences. Furthermore, the treatment appeared to be specific for the *CLCN7* mRNA because we did not observe any off-target effect in femurs on *CLCN3* and *CLCN5* transcripts (**Supplementary Figure S6**).

### Bone phenotype

**Figure 3:**
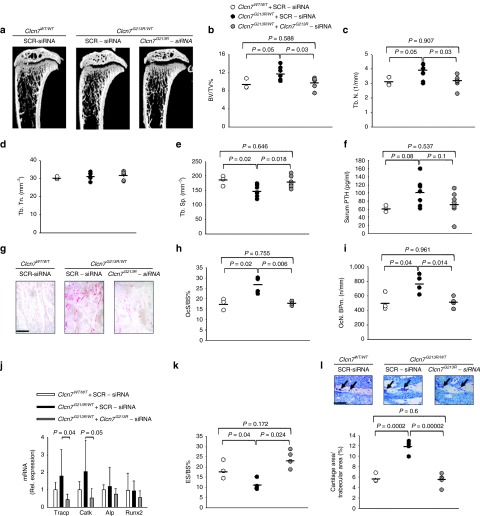
**Rescue of the bone phenotype**. Ten-day-old *Clcn7*^*WT/WT*^ and *Clcn7*^*G213R/WT*^ were treated with 4 mg/kg of scrambled- (SRC) or *Clcn7*^*G213R*^-sticky siRNA jetPEI conjugate, three times a week for 4 weeks. At the end of the experiments, mice were sacrificed and their bone phenotype analyzed. (**a**) µCT analysis of proximal tibias. (**b**) Trabecular bone volume over total tissue volume (BV/TV). (**c**) Trabecular number (Tb.N). (**d**) Trabecular thickness (Tb.Th). (**e**) Trabecular separation (TB.Sp). (**f**) Serum concentration of ParaThyroid Hormone (PTH). (**g**) Histochemical TRAcP staining to evaluate osteoclasts (purple cells). Bar = 100 µm. (**h**) Osteoclast surface over bone surface (Oc.S/BS). (**i**) Osteoclast number over bone perimeter (Oc.N/B Pm). (**j**) Transcriptional expression, by real-time RT-PCR on RNA extracted from the whole femurs of osteoclast (*Tracp* and *Cathepsin K* (*CatK*)) and osteoblast (*Alkaline phosphatase* (*ALP*) and *Runt-related transcription factor 2* (*Runx 2*)) genes normalized with *gapdh*. (**k**) Eroded surface over bone surface (ES/BS). (**l**) Representative images of the secondary spongiosa (upper panels) and measurement of cartilage area/trabecular area (lower panel). Arrows: cartilage remnants. Bar = 50 µm. Results are (**a,g,i** (upper panels)) representative or (**b–f,h–i** (lower panel)) the mean ± SD of three to seven mice/group (Student's *t*-test). In **d**, *P* > 0.2. For **b–f,h–l** statistics was also performed by one-way analysis of variance (shown in **Supplementary Table S3**).

To assess the bone phenotype of *Clcn7*^*G213R*^-sticky siRNA/jetPEI-treated ADO2 mice, we first evaluated the serum level of the bone resorption marker collagen type I cross-linked C-telopeptide (CTX) over the osteoclast marker Tartrate-Resistant Acid Phosphatase (TRAcP) 5b isoform, and observed a significant increase compared to scrambled sticky siRNA/jeiPEI conjugate-treated *Clcn7*^*G213R/WT*^ mice, peaking after 2 weeks of treatment (**Figure 2d**), which suggested activated bone resorption. After 4 weeks, serum CTX concentration returned to lower levels, probably due to the reduced number of osteoclasts indicated by the decreased levels of TRAcP (**Figure 2d**). These results suggest normalization of the osteoclast number, that in the *Clcn7*^*G213R/WT*^ mice was higher than in the *Clcn7*^*WT/WT*33^ (see **Figure 3g**–**i**), and re-establishment of normal bone resorption.

**Figure 4:**
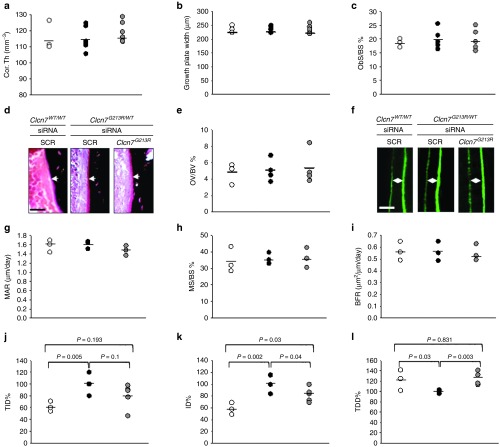
**Cortical, growth plate, osteoblast, dynamic, and bone quality variables**. Ten-day-old *Clcn7*^*WT/WT*^ and *Clcn7*^*G213R/WT*^ were treated with 4 mg/kg of scrambled- (SRC) or *Clcn7*^*G213R*^-sticky siRNA jetPEI conjugate, three times a week for 4 weeks. At the end of the experiments, mice were sacrificed and their bone phenotype analyzed. (**a**) Cortical thickness (Cor.Th). (**b**) Growth plate width. (**c**) Osteoblast surface over bone surface (Ob.S/BS). (**d**) Histological images of osteoid (arrows). Bar = 5 µm. (**e**) Osteoid volume over bone volume (OV/BV). (**f**) Calcein labeling (green fluorescence) of mineral deposition (double arrowheads). Bar = 2 µm. (**g**) Mineral apposition rate (MAR). (**h**) Mineralized surface over bone surface (MS/BS). (**i**) Bone formation rate (BFR). (**j**) Total indentation distance (TDI). (**k**) First-cycle indentation distance (ID). (**l**) Touchdown distance (TDD). Results are (**d,f**) representative or (**a–c,e,g–l**) the mean ± SD of three to seven mice/group (Student's *t*-test). In **a–c,e,g–i**, *P* > 0.2. For **a–c,e,g–l** statistics was also performed by one-way analysis of variance (shown in **Supplementary Table S3**).

After 2 weeks of treatment, µCT analysis of proximal tibias of *Clcn7*^*G213R/WT*^ mice treated with *Clcn7*^*G213R*^-sticky siRNA/jetPEI showed an improvement of the trabecular bone (**Figure 2e**) with significant decrease of trabecular bone volume over total tissue volume (**Figure 2f**), associated with decrease of trabecular number and thickness, and increase of trabecular separation (**Figure 2g**–**i**). These variables were further improved after 4 weeks of treatment, when we observed full rescue of the bone phenotype, with trabecular bone structural variables returning to the levels of wild-type mice (**Figure 3a**–**e**). Treated mice also showed a trend of reduction of ParaThyroid Hormone (PTH) (**Figure 3f**), decrease of osteoclast number over bone perimeter and osteoclast surface over bone surface (**Figure 3g**–**i**), and reduction of mRNA expression of osteoclast-specific genes (**Figure 3j**), which are in agreement with the reduced serum levels of TRAcP (**Figure 2d**). Importantly, bone resorption was increased, as demonstrated by the larger erosion surface (**Figure 3k**) and the reduced trabecular cartilage remnants in the secondary spongiosa (**Figure 3l**) observed by histomorphometry. *Clcn7*^*G213R/WT*^ mice showed no alteration of cortical bone (**Figure 4a**), growth plate (**Figure 4b**), femur length (**Supplementary Figure S7**), osteoblast (**Figure 4c**), osteoid (**Figure 4e**), and dynamic (**Figure 4f**–**i**) variables compared to *Clcn7*^*WT/WT*^ mice, and these features were not affected by the treatment, suggesting no direct effect of *Clcn7*^*G213R*^-sticky siRNA/jetPEI on bone formation, nor impairment of osteoclast-to-osteoblast crosstalk. Finally, the evaluation of indentation properties demonstrated a tendency of reduction of total indentation distance, significant reduction of first cycle indentation distance (**Figure 4j**,**k**) and significant increase of touchdown distance (**Figure 4l**), suggesting improvement of the biomechanical quality of the bones collected from the *Clcn7*^*G213R*^-sticky siRNA/jetPEI-treated *Clcn7*^*G213R/WT*^ mice. Taken together, these results suggest a successful *in vivo* experimental therapy for *Clcn7*^*G213R/WT*^ ADO2 mice with *Clcn7*^*G213R*^-specific siRNA.

### Treatment with human-specific siRNAs

**Figure 5:**
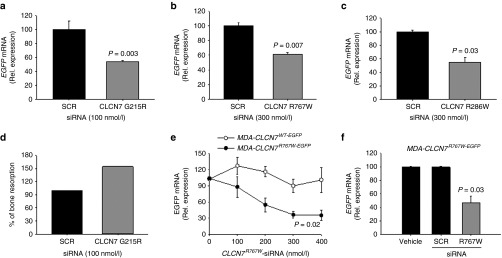
**Treatment of human cells**. Human osteoclasts transfected with the indicated expression vectors were treated for 48 hours with the indicated concentrations of (**a**) *Clcn7*^*G215R*^-, (**b**) *Clcn7*^*R767W*^-, and (**c**) *Clcn7*^*R286W*^-specific siRNAs. Real-time RT-PCR using primer pairs specific for *EGFP*, normalized with *GAPDH*. (**d**) Osteoclasts were generated from the peripheral blood mononuclear cells of *CLCN7*^*G215R/WT*^ ADO2 patient onto bone slices and treated with the indicated concentration of SCR and *Clcn7*^*G215R*^-specific siRNA. At the end of experiment, cells were removed by sonication and bone resorption evaluated by the pit assay. (**e**) Human breast cancer cells MDA-MB-231 (MDA) were transfected with *Clcn7*^*WT-EGFP*^ or *Clcn7*^*R767W-EGFP*^ and treated for 96 hours with the indicated concentrations of *Clcn7*^*R767W*^-specific siRNA. Real-time RT-PCR using primer pairs specific for *EGFP*, normalized with *GAPDH*. (**f**) Balb/c *nu/nu* atymic mice were subcutaneously inoculated in both flanks with MDA cells transfected with *Clcn7*^*R767W-EGFP*^ expression vector. When tumors reached the volume of 1 cm^3^, animals were treated with 4 mg/kg body weight of *Clcn7*^*R767W*^-specific siRNA. After 96 hours, tumors were excised, RNA extracted, and evaluated for *Clcn7*^*R767W*^ expression by real time RT-PCR, using primer pairs specific for *EGFP*, normalized with *GAPDH*. Results are the mean ± SD of (**d**)one single experiment, (**a-c,e**) three independent experiments and (**f**) three mice/group. **a–c,f**: Student's *t*-test; **e**: one-way analyis of variance.

*In vitro* treatment of human blood-derived osteoclasts, transfected with vectors carrying the *CLCN7*^*G215R-EGFP*^, the *CLCN7*^*R767W-EGFP*^, and the *CLCN7*^*R286W-EGFP*^ mutants, with *CLCN7*^*mutant*^-specific siRNAs efficiently reduced all *CLCN7*^*mutant*^ mRNAs (**Figure 5a**–**c**), suggesting that the therapy could be suitable also for human cells. By chance, we had access to the peripheral blood mononuclear cells of a *CLCN7*^*G215R/WT*^ ADO2 patient, who presented with diffuse cranial and vertebral sclerosis, optic nerve compression, muscle hyposthenia, reduced red cell count, low hemoglobin and hematocrit, and increased creatine kinase. In one single experiment, we could assess the efficacy of the *CLCN7*^*G215R*^*-*specific siRNA on bone resorption. We observed an increase of the pit area over bone section area in the osteoclasts treated with the *CLCN7*^*G215R*^*-*specific siRNA versus osteoclasts treated with the scrambled siRNA (**Figure 5d**). Although there are important limitations in testing the treatment in a single osteoclast culture from a single patient, this result could strengthen the hypothesis that the therapy could improve bone resorption also in human ADO2 osteoclasts.

Finally, in order to assess whether the siRNA targeting the human mutant gene could be used *in vivo*, we tested an alternative human/mouse model. We generated subcutaneous human tumors in atymic mice upon inoculation of human breast cancer MDA-MB-231 cells transfected with the *CLCN7*^*R767W*^ vector. These cells were sensitive to *CLCN7*^*R767W*^-specific siRNA treatment *in vitro*, showing a specific concentration-dependent downregulation of *ClCN7*^*R767W-EGFP*^ chimeric mRNA (**Figure 5e**). Mice where then treated with a single i.p. injection of *CLCN7*^*R767W*^-specific siRNA, tumors were excided after 96 hours and examined for *CLCN7*^*R767W-EGPF*^ chimeric RNA expression by RT-PCR. In this circumstance, we observed lesser *CLCN7*^*R767W-EGPF*^ mRNA in tumors subjected to *CLCN7*^*R767W*^-specific siRNA treatment, with no effect induced by scrambled siRNA (**Figure 5f**).

## Discussion

Our study demonstrates the feasibility of an effective treatment of murine *CLCN7*-dependent ADO2. To the best of our knowledge, this is the first report that describes a specific experimental cure for this therapeutically neglected disease, opening up an avenue for future developments in humans.

The positive effects of siRNA treatment were already appreciable after 2 weeks, and after 4 weeks there was a rescue of the bone phenotype, which included normalization of osteoclast and bone resorption serum biomarkers, bone structural and osteoclast variables. Early detection of reduced serum TRAcP and increased CTX has a great translational relevance as these parameters could be used in patients to monitor the progression of the treatment. Notably, the reduced serum TRAcP level in treated mice indicates normalization of osteoclast number, confirmed also by histomorphometry, suggesting that the treatment also interferes with the pressure to osteoclastogenesis typical of ADO2. In fact, in our siRNA-treated mice, we observed a trend to decrease of PTH, known to be implicated in osteoclastogenesis true its ability to increase RANKL and reduce osteoprotegerin expression.^38^ The concomitant increase of serum CTX and of the CTX/TRAcP ratio suggest that in the treated mice there were less numerous but more active osteoclasts. This result, along with the increased erosion surface and the reduced trabecular cartilage remnants in secondary spongiosa, suggest that indeed the siRNA used in this study efficiently targeted the osteoclasts *in vivo*, directly improving their activity, while there was no evidence of siRNA effect on osteoclast formation.

Despite osteoblasts also express the *Clcn7* gene, albeit at a lower level compared to osteoclasts, the mutant gene did not affect bone formation.^34^ Consistently, treatment with siRNA was inactive on osteoblasts and on dynamic variables. This result also suggests no changes in the osteoclast-osteoblast cross talk, which could increase bone formation. Although we have no experimental evidence explaining this observation, speculatively we could hypothesize that 4 weeks of treatment may not be long enough to affect this process.

In many ADO2 patients, the quality of life is extremely compromised especially due to frequent bone fractures, osteomyelitis and, in severe forms, hematological impairments and nerve compression syndromes.^34,39^ Some forms appear very early in life and, due to the severity of their symptoms, in the absence of a genetic analysis, they were incorrectly diagnosed as autosomal recessive osteopetrosis and successfully treated with hematopoietic stem cells transplantation.^40^ However, because of the intrinsic high risk of the procedure, this therapeutic option is not generally considered for most ADO2 forms, that become apparent later in life and have a milder course.^31^ Nevertheless, patients still suffer lifelong of severe symptoms and nowadays can be treated only palliatively.^32^ Recent preclinical treatment, partially improving the bone phenotype, has been demonstrated for interferon-γ, a cytotoxic and immunomodulator agent.^41^ However, interferon-γ is known to frequently cause important adverse effects, including suppression of bone marrow function, flu-like symptoms, diarrhea, fatigue, reduced ability to fight infections, confusion, etc., which are not expected in our study, considering the specificity of our targeted therapy.

Our results clearly show that our *Clcn7*^*G213R*^-specific siRNA can prevent the increase of bone mass in growing ADO2 mice. It will be necessary to test the therapy in adult and ageing mice to ascertain its efficacy also in reducing the bone mass later in life. Our preliminary results, obtained injecting 3-month-old mice with *Clcn7*^*G213R*^-specific siRNA at the same regimen used in this study, show that indeed this is the case. However, further work is necessary to consolidate this observation.

siRNA specificity allows the suppression of virtually all genes, provided that they are correctly designed. Somatic mutations in cancer^13^ and germline missense dominant negative mutations^14^ are considered excellent targets for siRNA silencing. However, mRNAs with single nucleotide mutations, as those generally observed in genetic dominant diseases, are difficult to eradicate, and “trial and error” strategies are generally used to identify effective and highly specific siRNA candidates. This has been done in our study, taking advantage of previous reports showing that additional nucleotide mismatches could allow satisfactory discrimination between mutant and normal mRNAs.^23^ In fact, we have obtained a series of siRNAs specific and effective for three human *CLCN7* ADO2 mutations, one of which could be tested also in the only available *Clcn7*-dependent ADO2 mouse model. Our study confirmed that the systemic delivery is efficient when our siRNA is modified by 3' dAdT overhangs and conjugated with the PolyPlus Transfection jetPEI linear polyethylenimine derivative. Improved stability and distribution, as well as prevention of immune and inflammatory responses to the siRNA, are important features to be considered for *in vivo* treatments, and our strategy appeared fully successful in this respect. We observed no adverse events in our mice, or signs of sufferance or distress during the entire length of our treatment.

In our study, we did not observe off-target effects of *Clcn7*^*G213R*^ siRNA on *Clcn3* and *Clcn5* transcripts, suggesting high specificity of the proposed treatment. However, we could not address specificity against a large part of the genome, therefore further work is necessary to rule out any potential undesirable off-target influence. Nevertheless, since this is a concern for any type of RNA interference therapy, there are great attention and effort, especially by companies, to identify appropriate strategies, including chemical modifications and rational design filters, to overcome this potential problem.^42^

Further means to reduce off-target effects could be to specifically deliver the siRNA to the osteoclasts, which represent that main cell type implicated in ADO2. Associations of siRNA to liposomes, nanoparticles, exosomes and other types of extracellular vesicles could be considered promising targeted delivery strategies if they could be conjugated with highly specific osteoclast-seeking factors. However, due to the wide distribution of the *CLCN7* gene expression, we believe that targeting multiple cell types could only be beneficial to improve the phentoype if the siRNA knocks down exclusively the target gene.

siRNA therapy is believed to become the gold standard treatment for certain diseases.^23^ Clinical trials are currently ongoing for a number of pathologies,^8^ with a more rapid progress for those with a local extension, such as ocular impairments.^43^ No doubt, however, that there is a great interest to develop systemic treatments for which siRNAs rather than shRNAs are probably more suitable.^10^ In fact, shRNAs entail the diseased cells to be transduced to express the silencing molecule endogenously, a condition that typically requires gene-like therapy.^9^ Although gene therapy is developing fast in recent years, it is still confined to selected genetic diseases especially characterized by immunodeficiency. siRNA therapy would instead have the advantage to be injectable by standard methods and be theoretically suitable for systemic delivery. In our experimental setting, the duration of gene silencing is 48 hours, which implies frequent redosing to maintain stable knock down. To reduce this burden, siRNAs should be made stable with a pharmacokinetics appropriate for a prolonged use. Considering the current great effort to obtain these conditions,^12,18,44^ we are confident that future developments will allow an easy use of siRNA also for systemic human diseases.^45^

Long-term systemic injectable therapies have the disadvantage to reduce compliance. Furthermore, long-term adverse effects are currently unpredictable because longitudinal clinical studies must be accumulated over the years to address this concern. However, it is possible to hypothesize that in *CLCN7*-dependent ADO2, the treatment could be compulsory especially during the growth phase, when bone modeling and remodeling are very active and osteoclast bone resorption is essential for the proper accrual, shaping, and homeostasis of the skeleton.^46^ In adulthood, bone remodeling serves especially for preserving a healthy bone mass. Therefore, we can predict that siRNA treatment could be lessened or given discontinuously, with a better compliance. To date, we are not able to make any speculation on this specific aspect. However, other long-term injectable treatments, such as insulin therapy for diabetes, are already in use for millions of patients, allowing us to predict that it will not be difficult to adjust the siRNA treatments also for the long-term use.

In conclusion, this work has demonstrated the feasibility of a siRNA-based therapy for *Clcn7*-dependent ADO2, opening an avenue for the cure of this disease. Further studies are now necessary to consolidate this knowledge and translate the results into benefits for patients.

## Materials and methods

*Materials.* Dulbecco's modified minimum essential medium (DMEM), fetal bovine serum, penicillin, streptomycin, and trypsin were from GIBCO (Uxbridge, UK). Sterile plastic ware was from Falcon Becton-Dickinson (Cowley, Oxford, UK) or Costar (Cambridge, MA). Trizol reagent, primers, and reagents for RT-PCR were from Invitrogen (Carlsbad, CA). The Brilliant SYBR Green QPCR master mix was from Stratagene (La Jolla, CA). Human recombinant (hr) Receptor Activator of Nuclear Factor κ-light-chain-enhancer of activated B cells transcription factor Ligand (RANKL) (#310-01) and hrMacrophage-Colony Stimulating Factor (M-CSF) (#300–25) were from Peprotech (EC, London). The cationic polymer transfection reagent *in vivo*-jetPEI (cat# 201-50G) was from Polyplus-transfection (Illkirch, France). Scrambled siRNA and siRNAs specific for all the *CLCN7* mutants were purchased by GE Dharmacon (Lafayette, CO). The Mouse TRAcP 5b isoform kit and the RatLaps EIA kit for CTX detection and bovine bone slices (cat. N. DT-1BON1000-96) were from Immunodiagnostic Systems (Gaithersburg, MD). Mouse PTH 1–84 ELISA kit (cat.#60–2305) was from Immunotopics (San Clemente, CA). The Reflotron kits were from Roche Diagnostics (Manheim, Germany). All the other reagents were of the purest grade from Sigma Aldrich (St. Louis, MO).

*Animals.* Procedures involving animals and their care were conducted in conformity with national and international laws and policies (European Economic Community Council Directive 86/609, OJ L 358, 1, December 12, 1987; Italian Legislative Decree 4.03.2014, n.26, Gazzetta Ufficiale della Repubblica Italiana no. 61, March 4, 2014).

*Cells lines.* The Human Embryonic Kidney 293 (HEK293), the human breast cancer MDA-MB-231, and the mouse leukemic monocyte-macrophage RAW264.7 cell lines were obtained from the American Tissue Culture Collection (ATCC, Rockville, MD) and grown in DMEM supplemented with 10% fetal bovine serum, 100 IU/ml penicillin, 100 μg/ml streptomycin, and 2 mmol/l L-glutamine, in a humidified 95% air/5% CO_2_ incubator at 37 °C.

*Osteoclast primary cultures.* Bone marrow flushed out from the bone cavity of the long bones of 7-day-old mice (C57BL6/J background) was diluted 1:1 in Hank's balanced salt solution, layered over Histopaque 1077 solution and centrifuged at 400*g* for 30 minutes. Cells were washed twice with Hank's solution, resuspended in DMEM, and plated in culture dishes at a density of 10^6^ cells/cm^2^. After 3 hours, cultures were rinsed to remove nonadherent cells and maintained for 7 days in the same medium supplemented with 50 ng/ml rhM-CSF and rhRANKL (120 ng/ml).

Human osteoclasts were differentiated from the peripheral blood mononuclear cells. Diluted blood (1:1 in Hanks' solution) was layered over Histopaque 1077 solution and centrifuged at 400*g* for 30 minutes. Buffy-coat cells thus isolated were washed twice with Hanks' solution, resuspended in DMEM containing 10% fetal bovine serum, and plated in culture dishes at a density of 10^6^ cells/cm^2^. After 3 hours, cultures were rinsed to remove nonadherent cells and incubated for 2 weeks in the same medium, in the presence of 50 ng/ml M-CSF and 30 ng/ml RANKL.

*In vitro bone resorption assay.* Osteoclasts were differentiated as described above but onto bovine bone slices. Slices were cleaned free of cells and stained with 1% toluidine blue. The pit area was then computed and expressed as % of bone resorption.

*Stable transfection.* The pEGFP-C1 plasmid was used because it allowed the generation of a chimeric *CLCN7-EGFP* transgene that could be identified through the *EGFP* tag. The vector backbone also contained a neomycin resistance cassette, used for the selection of stably transfected cells. HEK293 and RAW264.7 cells were stably transfected with vectors containing the *CLCN7* wild type (*CLCN7*^WT^) or the following *CLCN7* mutants: *CLCN7*^*G215R*^, *CLCN7*^*R286W*^, and *CLCN7*^*R767W*^, using the lipofectamine reagent. These cells were used for *in vitro* siRNA screening.

MDA-MB-231 cells were stably transfected with vectors containing the *CLCN7* wild-type (*CLCN7*^WT^) or the *CLCN7*^*R767W*^ mutant. They were used to establish *in vivo* tumors in mice and test a siRNA specific for the mutant, for which there are no ADO2 mouse models.

*Amaxa nucleofector transfection.* 1 × 10^6^ primary human osteoclasts were nucleofected with 2 μg of empty- or *CLCN7*^*WT*^, *CLCN7*^*G215R*^, *CLCN7*^*R286W*^, and *CLCN7*^*R767W*^-carrying pEGFP.C1/*EGFP* expression vectors, using the Amaxa Human Macrophage Nucleofector kit (Cat.# VPA-1008, Lonza, Cologne, Germany) and the program Y-010 of the nucleofector device.

*Conventional and real-time RT-PCR.* Total RNA was extracted using the Trizol procedure. RNA (1 μg) was reverse transcribed in cDNA using M-MLV reverse transcriptase and the equivalent of 0.1 μg was employed for the real-time PCR reactions using the Brilliant SYBR Green QPCR master mix or for conventional PCR. Results, expressed as fold increase for real time RT-PCR, or shown by electrophoresis of PCR products in a 2% agarose gel plus ethidium bromide for conventional RT-PCR, were normalized versus the housekeeping gene *Gapdh*.

*In vivo treatment.* Ten-day-old *Clcn7*^*G213R/WT*^ male mice were treated with *Clcn7*^*G213R*^-sticky siRNA (4 mg/kg) conjugated with *in vivo* jetPEI, i.p., three times a week for 2 and 4 weeks. In a pivotal experiment, 10-day-old *Clcn7*^*G213R/WT*^ mice were treated once with 2 and 4 mg/kg of *Clcn7*^*G213R*^-sticky siRNA jetPEI conjugated and sacrificed after 48 hours. Control groups included *Clcn7*^*G213R/WT*^ and *Clcn7*^*WT/WT*^ mice treated with jetPEI-conjugated sticky scrambled-siRNA. The jetPEI solution and the siRNAs were dissolved in 100–150 μl of ddH_2_O supplemented with 5% glucose, according to the manufacturer's instructions.^39^

*Subcutaneous tumor cell injection.* Four-week-old female Balb/c^*nu/nu*^ mice were anesthetized with ketamine and xylazine (75 + 15 mg/kg, respectively) i.p., then MDA-MB-231 cells stably transfected with the pEGFP.C1/*EGFP* plasmid carrying the *CLCN7*^*R767W*^ mutant gene were injected (1 × 10^6^/50 μl PBS) subcutaneously into both flanks using a tuberculin syringe with a 27½G needle (number of animals/group = 3). When the tumors reached a volume of 1 cm^3^, mice were treated with jetPEI conjugated with scrambled siRNA or with siRNA specific for the *CLCN7*^*R767W*^ mutation. After 96 hours, mice were sacrificed; tumors were excised and subjected to RNA extraction.

*Serum biomarkers.* Mouse serum levels of CTX, TRAcP 5b isoform, and PTH were measured by ELISA kits according to the manufacturer's instructions. Biomarkers of diseases were measured using the Reflotron kits.

*Micro computed tomography (μCT) analysis.* Images from tibias fixed in 4% formaldehyde were acquired in a SkyScan 1174 with a resolution of 6 μm (X-ray voltage 50 kV). Image reconstruction was carried out employing a modified Feldkamp algorithm^47^ using the Skyscan Nrecon software. 3D and 2D morphometric parameters were calculated for the trabecular bone (350 consecutive slides, 6 μm thick) starting 300 µm from the growth plate. The cortical thickness was analyzed in 100 consecutive slides, 6 μm thick, starting 2.1 mm from the growth plate. Threshold values were applied for segmenting trabecular bone corresponding to bone mineral density values of 0.6/cm^3^ calcium hydroxyapatite. 3D parameters were based on the analysis of a Marching Cubes type model with a rendered surface.^48^ Calculation of all 2D areas and perimeters was based on the Pratt algorithm.^49^ Bone structural variables and nomenclature were those suggested in Bouxsein *et al*.^50^

*Bone histomorphometry.* Tibias fixed in 4% paraformaldehyde were dehydrated in ethanol and processed for methacrylate embedding without decalcification. Histomorphometric measurements were carried out on 5-µm thick sections with an interactive image analysis system (IAS 2000; Delta Sistemi, Rome, Italy)^51^ and with the suggested nomenclature.^52^ The analysis was peformed in the proximal tibia trabecular region, 50 µm inside the cortical bone 100–450 µm below the growth plate. For the cartilage remnants, the analysis was performed in the secondary spongiosa, 450–650 µm below the growth plate and 50 µm inside the cortical bone. Osteoclast number/bone perimeter (number/mm) and osteoclast surface/bone surface (%) were evaluated after histochemically staining the sections for TRAcP activity. Osteoblast surface/bone surface (%) was evaluated after staining the sections with toluidine blue, while dynamic assessment of the mineral apposition rate was calculated after double injection of calcein, 10 and 3 days before sacrifice. Bone formation rate was calculated according to the following formula: mineral apposition rate × mineralized surface/bone area as suggested by Dempster *et al*.^52^

*Statistics.* Results are expressed as the mean ± SD of at least three independent experiments or mice/group. Statistical analyses were performed by the Student's *t*-test or the one-way analysis of variance, according to the type of data sets. Statistical methods are indicated in the figure legends and in **Supplementary Table S3**. A *P* value of <0.05 was conventionally considered statistically significant.

**SUPPLEMENTARY MATERIAL** **Table S1.** ADO2 CLCN7 gene mutations known to date^31,32,34,37,38^. **Table S2.** Primer pairs specific for *Clcn7*^*G213R*^ mRNA and PCR conditions. **Table S3.** Statistical analysis by one way ANOVA or one way ANOVA on ranks (*) of three-point data sets. **Figure S1.** Effect of *CLCN7*^mutant^-specific siRNAs on *CLCN7*^mutant^ knock down in RAW264.7 cells. **Figure S2.** Specificity of *Clcn7*^G213R^-siRNA. **Figure S3.** Osteoclastogenesis assay. **Figure S4.** Cellular expression of *Clcn7*. **Figure S5.** Comparative mRNA expression of *Clcn7* in mouse osteoclasts and osteoblasts. **Figure S6.** Effect of *Clcn7*^G213R^-specific siRNA/jetPEI complex on *Clcn3* and *Clcn5* gene expression. **Figure S7**. Femur length.

## Figures and Tables

**Table 1:**
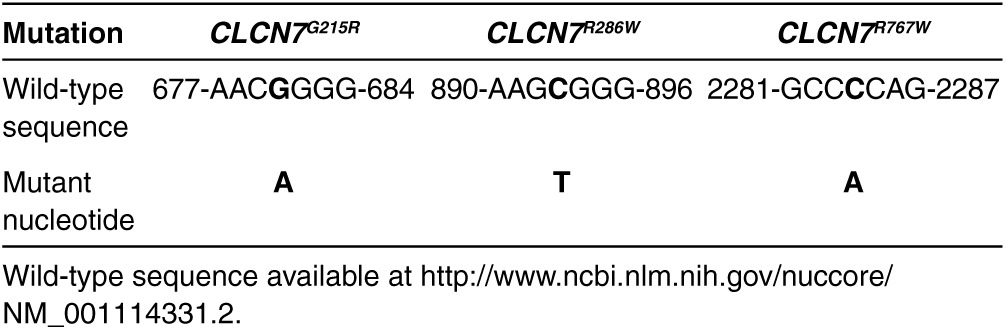
Nucleotide sequences of the wild-type *CLCN7* gene at the sites of each mutation and the corresponding mutant nucleotide as derived from direct DNA sequencing of the mutant constructs

**Table 2:**
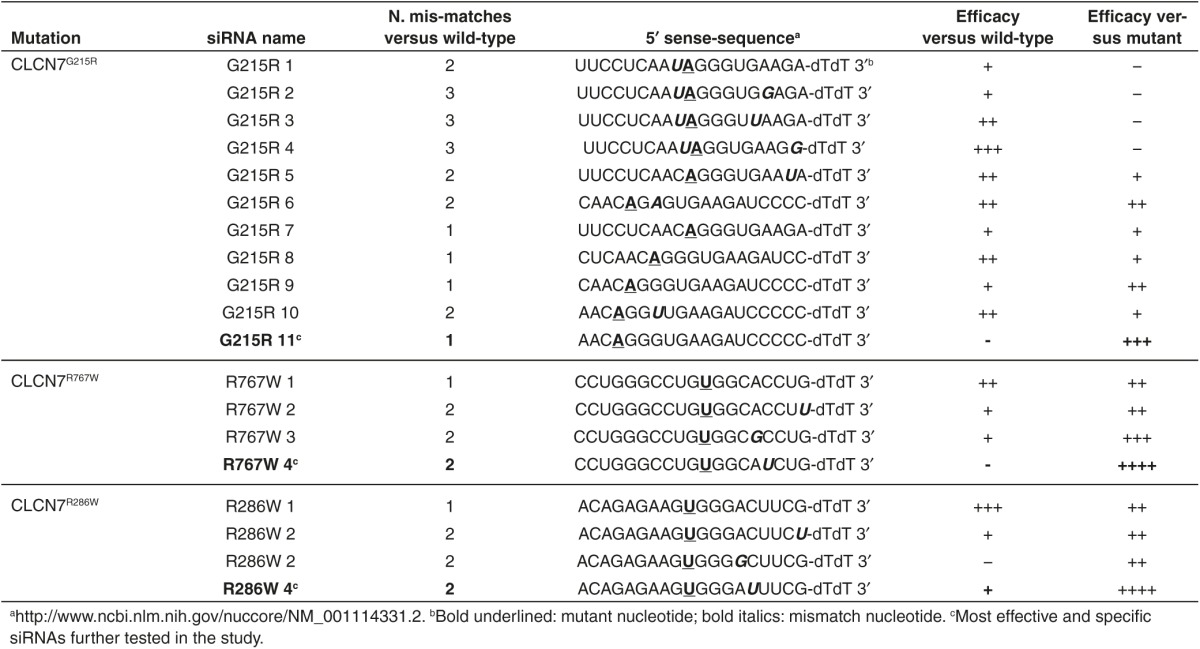
List of siRNAs designed and tested in the study

**Table 3:**
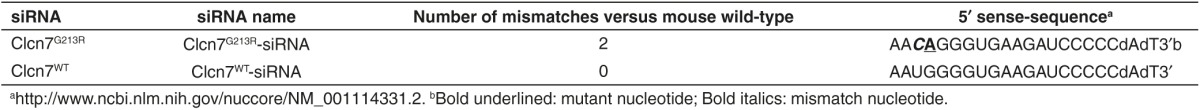
*Clcn7*^*G213R*^-siRNA sequence and mismatch nucleotides compared to mouse *Clcn7*^*WT*^ sequence
