# Supplementary material for: Effective Small Interfering RNA Therapy to Treat CLCN7-dependent Autosomal Dominant Osteopetrosis Type 2
Source: Mol Ther Nucleic Acids. 2015 Sep 1;4(9):e248–. doi: 10.1038/mtna.2015.21 (PMC4877447; doi:10.1038/mtna.2015.21)
Supplement: Supplementary Figures and Tables [file mtna201521x1.doc]

SUPPLEMENTARY METHODS

**Bioinformatics analysis of CLCN7 expression in primary cells**

CLCN7 transcriptional levels were evaluated in various cell types, in primary cultures or isolated by FACS, using the Genevestigator V3 software^1^ (<https://genevestigator.com/gv/>). Data are from two datasets: i) HS-SAMPLES-0, comprising 369 arrays Human 133-2: Affymetrix Human Genome U133 Plus 2.0, and ii) MM-SAMPLES-0, comprising 320 arrays Affymetrix Mouse Genome 430 2.0. Results are shown as logarithm of fluorescence intensity of the microarray probe. According to the software output, logarithm levels 6.5-8.5 are considered low expression, 8.5-11.5 are considered medium expression, and 11.5-13.0 are considered high expression.

**Supplementary reference**

1. Hruz, T, Laule, O, Szabo, G, Wessendorp, F, Bleuler, S, Oertle, L, et al. (2008) **GENEVESTIGATOR V3: a reference expression database for the meta-analysis of transcriptomes.** Advances in Bioinformatics 2008: 420747.

SUPPLEMENTARY TABLES

**Table S1:** ADO2 *CLCN7* gene mutations known to date^31,32,34,37,38^. In bold the mutations for which siRNAs were designed and tested in this work.

| **ClC-7 mutations** | **Amino acid change** | **ClC-7 mutations** | **Amino acid change** |
| --- | --- | --- | --- |
| Y99C | Tyrosine to Cysteine | R286Q | Arginine to Glutamine |
| D145G | Aspartic acid to Glycine | P470Q | Proline to Glutamine |
| W179X | Tryptophan to Unknown | G677V | Glycine to Valine |
| G203D | Glycine to Aspartic acid | 688del | Amino acid deletion |
| L213F | Leucine to Phenilalanine | L490F | Leucine to Phenylalanine |
| **G215R** | **Glycine** to **Arginine** | K689E | Lysine to Glutamic acid |
| R409W | Arginine to Tryptophan | R762L | Arginine to Leucine |
| R409W | Arginine to Tryptophan | G765B | Glycine to Asparagine or Aspartic Acid |
| L490F | Leucine to Phenylalanine | L766P | Leucine to Proline |
| G677V | Glycine to Valine | **R767W** | **Arginine** to **Tryptophan** |
| P249L | Proline to Leucine | A788D | Alanine to Aspartic acid |
| **R286W** | **Arginine** to **Tryptophan** | 2423delAG | Adenine/Guanine deletion |

**Table S2**: Primer pairs specific for *Clcn7^G213R^* mRNA and PCR conditions

| **Primer pairs** | **Sequence** | **PCR conditions** |
| --- | --- | --- |
| *Clcn7^G213R^* | Fw: Caagtgcttcctcaatg  Rv: GCCCTCTTCCAAGCTAAA | 95° 10’  35 cycles 95° 30’’, 60° 30’’, 72° 30’’ |

**Table S3**: Statistical analysis by one way ANOVA or one way ANOVA on ranks (*) of three-point data sets.

| **Figure #** | **Clcn7^WT/WT^-SCR-siRNA**  **vs.**  **Clcn7^WT/G213R^-SCR-siRNA** | **Clcn7^WT/WT^-SCR-siRNA**  **vs.**  **Clcn7^WT/G213R^-Clcn7^G213R^-siRNA** | **Clcn7^WT/G213R^-Clcn7^G213R^-siRNA**  **vs.**  **Clcn7^WT/G213R^-SCR-siRNA** |
| --- | --- | --- | --- |
|  | **P-Value** | **P-Value** | **P-Value** |
| 1i | <0.05* | >0.1 | <0.05* |
|  |  |  |  |
| 2f | 0.001 | 0.027 | 0.017 |
| 2g | 0.001 | 0.005 | 0.04 |
| 2h | 0.018 | >0.1 | 0.08 |
| 2i | 0.011 | 0.017 | >0.1 |
|  |  |  |  |
| 3b | 0.06 | >0.1 | 0.08 |
| 3c | 0.1 | >0.1 | 0.07 |
| 3d | >0.1 | >0.1 | >0.1 |
| 3e | 0.04 | >0.1 | 0.04 |
| 3f | >0.1 | >0.1 | >0.1 |
| 3h | 0.007 | >0.1 | 0.009 |
| 3i | 0.03 | >0.1 | 0.03 |
| 3j (Trap) | >0.1 | >0.1 | <0.05* |
| 3j (Catk) | >0.1 | >0.1 | <0.05* |
| 3j (Alp) | >0.1 | >0.1 | >0.1 |
| 3j (Runx2) | >0.1 | >0.1 | >0.1 |
| 3k | 0.07 | >0.1 | 0.005 |
| 3l | 0.001 | 0.008 | 0.6 |
|  |  |  |  |
| 4a | >0.1 | >0.1 | >0.1 |
| 4b | >0.1 | >0.1 | >0.1 |
| 4c | >0.1 | >0.1 | >0.1 |
| 4e | >0.1 | >0.1 | >0.1 |
| 4g | >0.1 | >0.1 | >0.1 |
| 4h | >0.1 | >0.1 | >0.1 |
| 4i | >0.1 | >0.1 | >0.1 |
| 4j | 0.027 | >0.1 | >0.1 |
| 4k | 0.002 | 0.03 | 0.03 |
| 4l | >0.1 | >0.1 | 0.05 |
|  |  |  |  |
| S2 | >01* | >01* | >01* |
| S3 | >0.1 | 0.002 | 0.36 |
| S7 (*Clcn3*) | >0.6 | >0.6 | >0.6 |
| S7 (*Clcn5*) | >0.4 | >0.4 | >0.4 |
| S7 (*Clcn7^G213R^*) | 0.008 | 0.008 | 0.008 |

*One way ANOVA on ranks was applied when the data had no normal distribution.

SUPPLEMENTARY FIGURES

**Figure S1 – Effect of *CLCN7^mutant^*-specific siRNAs on *CLCN7^mutant^* knock down in RAW264.7 cells.** Murine leukemic monocyte-macrophage RAW264.7 cells, representing a model of osteoclast precursors, were transfected with the indicated vectors and treated for 48 hours with the concentrations of *CLCN7^G215R^*-, *CLCN7^R286W^*- and *CLCN7^R767W^*-specific siRNAs shown in *abscissa*. Real time RT-PCR using primer pairs specific for *EGFP*, normalized with *GAPDH*. Results are the mean±SD of 3 independent experiments (Student’s t test).

**Figure S2 – Specificity of *Clcn7^G213R^*-siRNA.** Osteoclasts generated from the bone marrow mononuclear cells of *Clcn7^WT/WT^* and *Clcn7^G213R/WT^* mice were treated with the indicated concentration of scrambled (SCR) or *Clcn7^G213R^*-specific siRNA. Real time RT-PCR was performed using primer pairs specific *Clcn3* and *Clcn5* mRNAs. Data are the mean±SD of 3 independents (Student’s *t* test).

**Figure S3 – Osteoclastogenesis assay.** Bone marrow mononuclear cells were isolated from *Clcn7^WT/WT^* and *Clcn^G213R/WT^* mice and subjected to osteoclastogenesis by treatment with M-CSF and RANKL, in the presence of scrambled (SCR)- or *Clcn7^G213R^*-specific siRNA as indicated. After 7 days, cultures were fixed and subjected to cytochemical staining of TRAcP, then TRAcP-positive multinuclear cells were enumerated. Data are the mean+S.D. of three independent cultures (Student’s *t* test). Statistics was also performed by ANOVA (shown in Table S3).

**Figure S4 *–*** **Cellular expression of Clcn7.** Transcriptional expression of Clcn7 evaluated using the Genevestigator V3 software (<https://genevestigator.com/gv/>), in databases of **(a)** human (HS-SAMPLES-0) and **(b)** mouse (MM-SAMPLES-0) primary cells. The Clcn7 expression is shown as logarithmic value of fluorescence intensity. **(c)** Immunohistochemical staining for ClC-7 in the indicated mouse organs. Bar = 25 µm. G: Glomerulus; T: Tubule; CV: central vein; H: hepatocytes; B: Bone; I: Pancreatic islet; A: Alveolus. Arrow: osteoblast; Arrowhead: osteoclast.

**Figure S5 – Comparative mRNA expression of Clcn7 in mouse osteoclasts and osteoblasts.** Real time RT-PCR of Clcn7 mRNA in mouse primary osteoclasts and osteoblasts, normalized with GAPDH. Results are the mean±SD of 3 independent experiments (Student’s t test).

**Figure S6 – Effect of Clcn7^G213R^-specific siRNA/jetPEI complex on Clcn3 and Clcn5 gene expression.** Ten day old *Clcn7^G213R/WT^* male mice, treated with 4 mg/Kg body weight with scrambled (SRC-) or *Clcn7^G213R^*-sticky siRNA/jetPEI, 3 times a week for 4 weeks, were sacrificed, RNA was extracted from femurs and subjected to RT-PCR with primer pairs specific for Clcn3, Clcn5 mRNAs. Clcn7^G213R^ mRNAs was amplified as positive control. Results are the mean+s.d of 3-6 mice/group (Student’s t test), p>0.2.

**Figure S7 – Femur length.** Ten day old *Clcn7^G213R/WT^* male mice, treated with 4 mg/Kg body weight with scrambled (SRC-) or *Clcn7^G213R^*-sticky siRNA/jetPEI, 3 times a week for 4 weeks, were sacrificed along with the WT littermates and subjected to X-ray analysis (36 kilovoltage per amperage, 10 sec.), then femur length was measured. Data are the mean+s.d of 3-5 mice/group (Student’s *t* test), p>0.2. Statistics was also performed by ANOVA (shown in Table S3).
